# QTL mapping of PEG-induced drought tolerance at the early seedling stage in sesame using whole genome re-sequencing

**DOI:** 10.1371/journal.pone.0247681

**Published:** 2021-02-24

**Authors:** Junchao Liang, Jian Sun, Yanying Ye, Xiaowen Yan, Tingxian Yan, Yueliang Rao, Hongying Zhou, Meiwang Le

**Affiliations:** 1 Crop Research Institute, Jiangxi Academy of Agricultural Sciences, Nanchang, China; 2 Nanchang Branch of National Center of Oilcrops Improvement, Nanchang, China; 3 Jiangxi Province Key Laboratory of Oilcrops Biology, Nanchang, China; 4 Horticulture Research Institute, Jiangxi Academy of Agricultural Sciences, Nanchang, China; Huazhong University of Science and Technology, CHINA

## Abstract

Improvement in sesame drought tolerance at seedling stage is important for yield stability. Genetic approaches combing with conventional breeding is the most effective way to develop drought-tolerant cultivars. In this study, three traits and their relative values, including seedling weight (SW), shoot length (SL) and root length (RL), were evaluated under control and osmotic conditions in a recombinant inbred line (RIL) population derived from cross of Zhushanbai and Jinhuangma. Significant variation and high broad sense heritability were observed for all traits except SW under stress condition in the population. With this population, a high-density linkage map with 1354 bin markers was constructed through whole genome re-sequencing (WGS) strategy. Quantitative trait loci (QTL) mapping was performed for all the traits. A total of 34 QTLs were detected on 10 chromosomes. Among them, 13 stable QTLs were revealed in two independent experiments, eight of them were associated with traits under water stress condition. One region on chromosome 12 related to RL under osmotic condition and relative RL had the highest LOD value and explained the largest phenotypic variation among all the QTLs detected under water stress condition. These findings will provide new genetic resources for molecular improvement of drought tolerance and candidate gene identification in sesame.

## Introduction

Drought stress usually refers to a water shortage which causes dramatic morphological, biochemical, physiological, and molecular changes [1]. These changes would severely affect plant growth and crop yield stability. In past four decades, it is estimated that the drought has caused a cereal loss of 1820 million Mg [2]. Meanwhile, the continuous global warming and climate change will probably increase the frequency of drought. Sesame (*Sesamum indicum* L., 2n = 26) is one of the most important oilseed crops in the world. Sesame seeds are known to be rich in protein, vitamins and special antioxidants, such as sesamin and sesamolin, which make sesame become a very healthy food favored by consumers. Comparison with most of other oilseed crops, sesame is considered as a resilient crop that is more tolerant to drought stress. However, sesame growth and productivity are vulnerable to severe drought stress, especially in the arid and semi-arid areas. Sesame belongs to shallow root plants and is very sensitive to drought stress during the germination and flowering stages [3]. Improvement of drought tolerance at these two stages of sesame is very important for yield stability.

Drought tolerance is a complex quantitative trait in plants. QTL mapping and genome-wide association study (GWAS) have been widely used for genetic analysis of drought tolerance related traits in many plants, such as rice [4,5], wheat [6–8], cotton [9,10] and *Brassica napus* [11]. These traits including root length, coleoptile length and shoot length for seedling stage, and yield-related traits for flowering stage. A solution of polyethylene glycol (PEG) is frequently used to simulate drought stress through treating the seeds with the PEG solution for days, especially in germination or seedling stress experiments [4–6,12,13].

In sesame, several studies have reported that many traits including germination rate, seedling growth, shoot length, root length and yield related traits could be affect by drought stress [14–16]. Mensah et al. [14] used varying PEG concentrations to simulate drought effect on germination of sesame and found that higher osmotic conditions (0.25–0.50 MPa) significantly reduced the germination rate, radical and shoot development, but lower osmotic tensions (0.0625 MPa) could enhance root growth. Only a few studies have been conducted yet on genetic analysis of drought tolerance. Li et al. [17] confirmed 15% PEG 6000 as a suitable concentration for examining drought tolerance in sesame germplasms, and performed a GWAS of stress tolerance indexes related to NaCl-salt and PEG-drought at the germination stage with 490 sesame accessions, and identified nine and 15 QTLs for drought and salt stresses, respectively. Ten stable QTLs were also identified for five drought related traits at the sesame flowering stage through GWAS [18]. By using gene association study, gene expression and transgenic experiments, a candidate gene *SiSAM* was identified to confer drought tolerance by modulating polyamine levels and ROS homeostasis in sesame [18]. So far, there is no study on QTL mapping for drought tolerance traits using bi-parental population in sesame.

The recent rapid development of high throughput sequencing makes it easier to construct high-density genetic map with numerous single-nucleotide polymorphism (SNP) markers. SNPs are the most abundant form of genetic variation throughout the genomes and are ideal genetic markers for genetic and breeding applications. Recent released sesame reference genome has greatly helped in SNPs identification in sesame through multiple next generation sequencing strategies, including genotyping by sequencing (GBS), reduced representation sequencing (RAD) and whole genome re-sequencing. These methods have been successfully utilized for high resolution mapping of QTLs in sesame [19–23].

To further explore the genetic foundation of sesame drought tolerance, in the present study, we developed a high-density genetic map through WGRS of 180 RILs generated by two sesame landraces Jinhuangma (JHM) and Zhushanbai (ZSB) from China. By performing PEG stress experiments, QTLs for fresh seedling weight, shoot length and root length were identified under control and stress conditions. These can provide a valuable contribution to understand the genetic basis of drought tolerance and facilitate marker-assisted breeding for stress tolerance in sesame.

## Materials and methods

### Plant materials and phenotyping

The *Sesamum indicum* L. cultivars Jinhuangma (JHM) and Zhushanbai (ZSB) were landraces collected at Jiangxi province and Hubei province respectively of China. JHM was more sensitive to drought than ZSB at seedling stage. The seeds of JHM and ZSB used in this study were obtained from sesame germplasm reservoir of Nanchang Branch of National Center of Oilcrops Improvement, Jiangxi Academy of Agricultural Sciences, China. A set of 180 F_9_ recombinant inbred lines developed by single seed descent from the cross of JHM and ZSB was used for QTL mapping. All the plants were grown in a nylon net house to prevent cross-pollination caused by insects.

Polyethylene glycol (PEG)-simulated drought stress trials were performed by using both parent and RIL population in two years. For each line, 50 mature seeds were germinated on two layers of filter paper in each plastic container (10 cm × 10 cm × 5 cm) with top. The plastic containers were maintained under dark conditions and a constant temperature of 28°C in climatic chamber for five days. For RIL population, two treatments were performed at the same time. In control condition, 40 mL ddH_2_O was added into each box while in PEG stress condition 40 mL of 15% w/v PEG6000/water solution was applied. Three independent biological replicates were performed for each genotype in both conditions. Two independent experiments were conducted in two years (2018 and 2019). 20 normal plants per genotype were phenotyped in each condition of each replication five days after germination. Data of root length (RL), shoot length (SL) and fresh seedling weight (SW) were recorded on individual plants. Drought tolerance index (DTI) was defined as relative traits value, which was estimated as the ratio of the traits value under PEG stress to the traits value under control condition. For each trial, all the phenotypic data were analyzed using the mean value of three replicates. Statistical analysis of phenotype data and Pearson correlation analysis were carried out using SPSS statistical package (SPSS Inc., Chicago, IL).

### DNA extraction, sequencing and SNP/InDel discovery

Total genomic DNA of JHM and ZSB along with 180 RILs were extracted from fresh young leaves following the standard protocols with the plant genomic DNA purification kit (B518261, Sangon Biotech Co., Ltd., Shanghai). Paired-end sequencing libraries with 300–500 bp insertion were constructed for each DNA samples. The libraries were sequenced with 150 bp (PE150) read length by using an Illumina Hiseq 2000 system (Illumina Inc., San Diego, CA, USA). The two parental genotypes were sequenced at ~10×depth, and individual lines were sequenced at ~3–6× depth coverage. The raw reads were filtered and aligned to the reference genome of sesame cultivar Zhongzhi No. 13 (Sinbase 2.0) [20] using the Burrows-Wheeler alignment (BWA) tool with default parameters [24]. SAMtools v1.9 [25] was used to convert sequence alignment map (SAM) format to binary alignment map (BAM) files. Aligned BAM files were sorted with SortSam in Picard (http://broadinstitute.github.io/picard/). SNP/InDel (Insertion and deletion) detection was performed by using HaplotypeCaller in Genome Analysis Toolkit (GATK) v4.0.11.0 [26].

### Linkage map construction

The SNPs or InDels were filtered according to three criteria: (1) missing data rate < 20%; (2) minor allele frequency (MAF) > 0.2; and (3) loci that were homozygous in both parents and heterozygous in less than 15% of the RILs. Filtered SNPs/InDels were used to construct bin-map through maximum parsimonious inference of recombination (MPR) method described by Xie et al. [27]. First, all variants were re-filtered by permutations involving resampling of SNPs/InDels windows and then inferred by Bayesian method. The genotype of each locus in RILs was determined assisted by hidden Markov model. Consecutive SNPs/InDels sites with the same genotype as one parent were assembled into a block. A recombination event was defined as a transition between two blocks with different genotypes. The R/qtl package (http://rqtl.org/) was used to construct the linkage map.

### QTL analysis

Analysis of variances (ANOVA) was performed to estimate the effects of genotype, environment and interaction between genotype and environment. The broad sense heritability was estimated with the formula: *H*^2^ = *σ*^2^_*g*_/(*σ*^2^_*g*_+*σ*^2^_*ge*_/*n*+*σ*^2^_*e*_/*nr*), where *n* is the number of environments, *r* is the number of replications, σ^2^_g_ is the estimated genetic variance, σ^2^_ge_ is variance for genotype-environment interaction and σ^2^_e_ is experimental error. QTLs for each trait in each experiment were identified by composite interval mapping (CIM) method using Window QTL Cartographer v2.5 [28] (http://statgen.ncsu.edu/qtlcart/WQTLCart.htm). The standard model 6 and a window size of 10 cM was applied. The level of significance was determined with 1000 permutations, with a confidence level of 95%. The LOD score for declaring a QTL was 2.5 or above. MapChart software was used to construct the graphical representation of QTL positions [29].

## Results

### Whole genome re-sequencing and genotyping

Using whole genome re-sequencing approach, we generated over 1.1 billion reads from two parents and 180 RILs, with an average of 6.3 million reads per RIL, providing an average read depth of 4.18×. For the two parents JHM and ZSB, ~14.6 million and ~13.9 million reads were obtained respectively. All the clean reads were mapped to the sesame reference genome Zhongzhi No. 13 [20] using BWA. After filtering, a total of 466,911 high-quality SNPs and 72,981 InDels were identified among the RILs. Chromosome (chr) 3 harbored the largest number of SNPs and InDels, while chromosome 7 contained the fewest number of variants. The density of the SNP and InDel loci in the genome was 1788.15/Mb and 280.32/Mb respectively (S1 Table).

### High density genetic map construction

**Fig 1:**
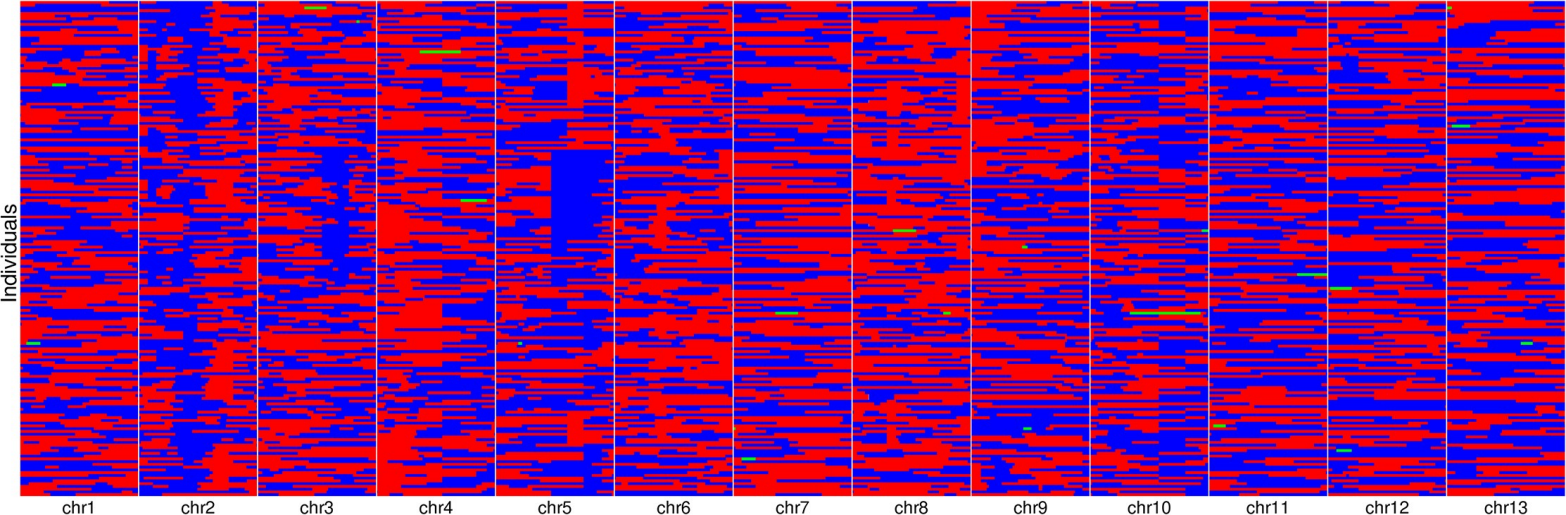
Genotype data of individuals of the RIL population. Red and blue represent Zhushanbai and Jinhuangma, green represents heterzygote.

**Fig 2:**
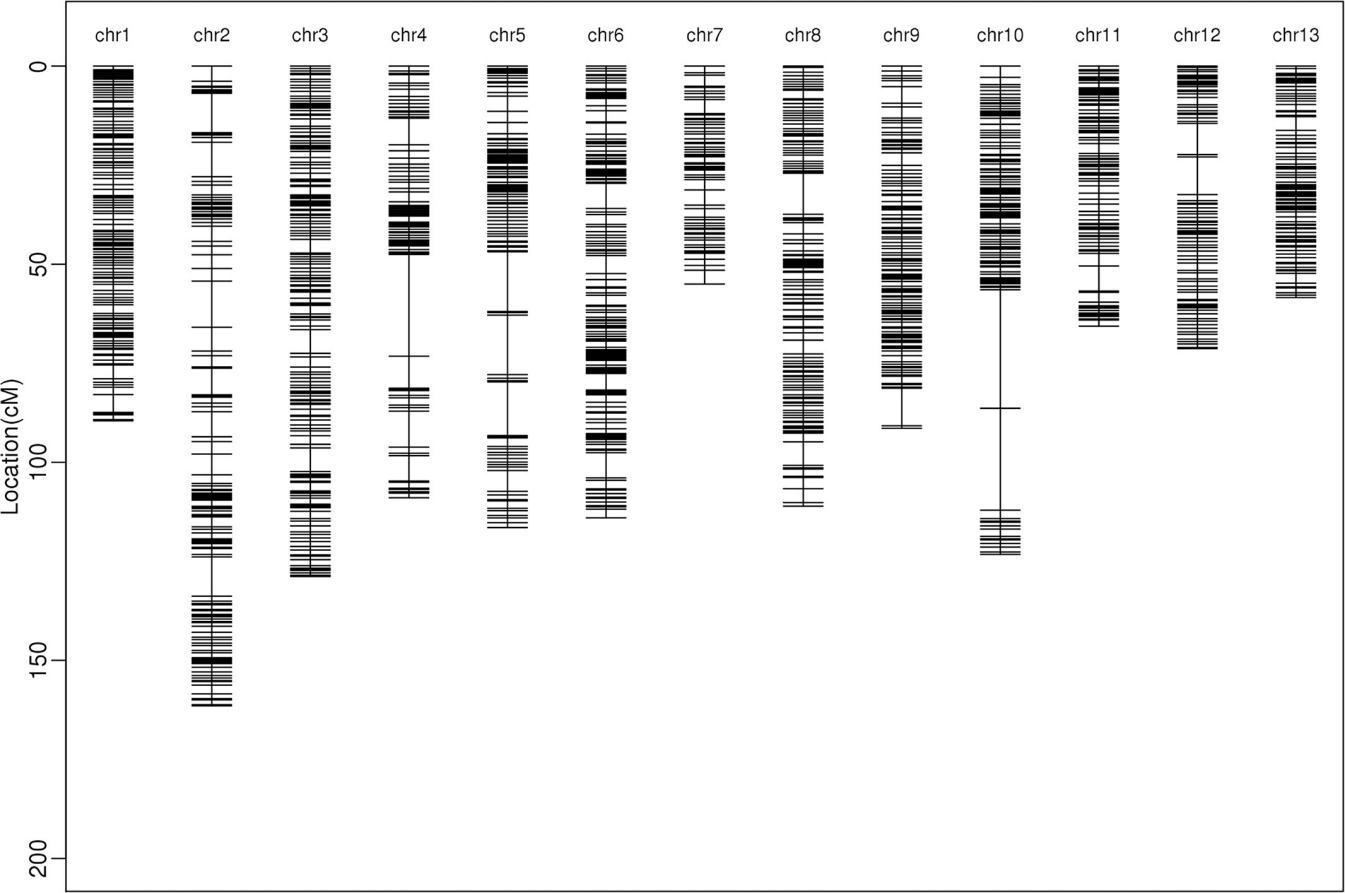
Distributions of bin markers on 13 chromosomes.

All the filtered SNPs/InDels were used to construct bin-map through MPR method. A total of 1354 bin markers covering 538,090 variants were identified on the 13 chromosomes (Fig 1). By using genotype data of the RIL population, we constructed a high-density genetic map with a total genetic distance of 1295.45 cM. The mapped bin per chromosome ranged from 58 (chr7) to 155 (chr3) with an average of 104.2 per chromosome (Table 1, Fig 2). The density of bin markers in the whole genome was 0.98 cM/locus, covering an average physical length of 158.74 kb per bin.

**Table 1:** Characteristics of the high-density genetic map.

Chr.^a^	Length(cM)	No. markers	No. bins	Average bin interval (cM)	Max interval (cM)
chr1	89.539	61646	125	0.722	4.528
chr2	161.486	38457	120	1.357	11.684
chr3	128.823	76256	155	0.837	5.969
chr4	108.981	26753	77	1.434	25.697
chr5	116.469	43314	97	1.213	15.049
chr6	114.018	60058	136	0.845	6.339
chr7	55.011	12058	58	0.965	3.833
chr8	111.070	45042	119	0.941	10.355
chr9	91.418	37416	114	0.809	9.503
chr10	123.251	36959	105	1.185	29.912
chr11	65.639	40345	85	0.781	6.339
chr12	71.325	31949	77	0.938	9.503
chr13	58.423	27837	86	0.687	3.491
Whole	1295.453	538090	1354	0.978	10.939

^a^Chr., Chromosome.

In addition to several gaps of more than 10 cM identified on chr2 (1), chr4 (1), chr5 (3), chr8 (1) and chr10 (2), the bin markers were distributed evenly along 13 chromosomes. The chromosome with the longest genetic length was chr2, which contained 120 bin markers covering a genetic length of 161.49 cM. Chr7 covered the shortest genetic length (55.01 cM) with 58 bin markers. A total of 1286 (95.0%) bin markers were less than 500 kb in length, and 40 bins covered physical length larger than 1 Mb. The largest bin located on chromosome 9 (c09b114), with a physical length larger than 5 Mb. (S2 Table).

To evaluate the quality of this high density genetic map, we investigated the collinearity between this genetic map and physical map. The dot plot of markers in the 13 linkage groups aligned well with the Zhongzhi No.13 reference chromosome, indicating excellent collinearity between genetic map and physical map (S1 Fig).

### Phenotypic variation and correlation analysis

**Fig 3:**
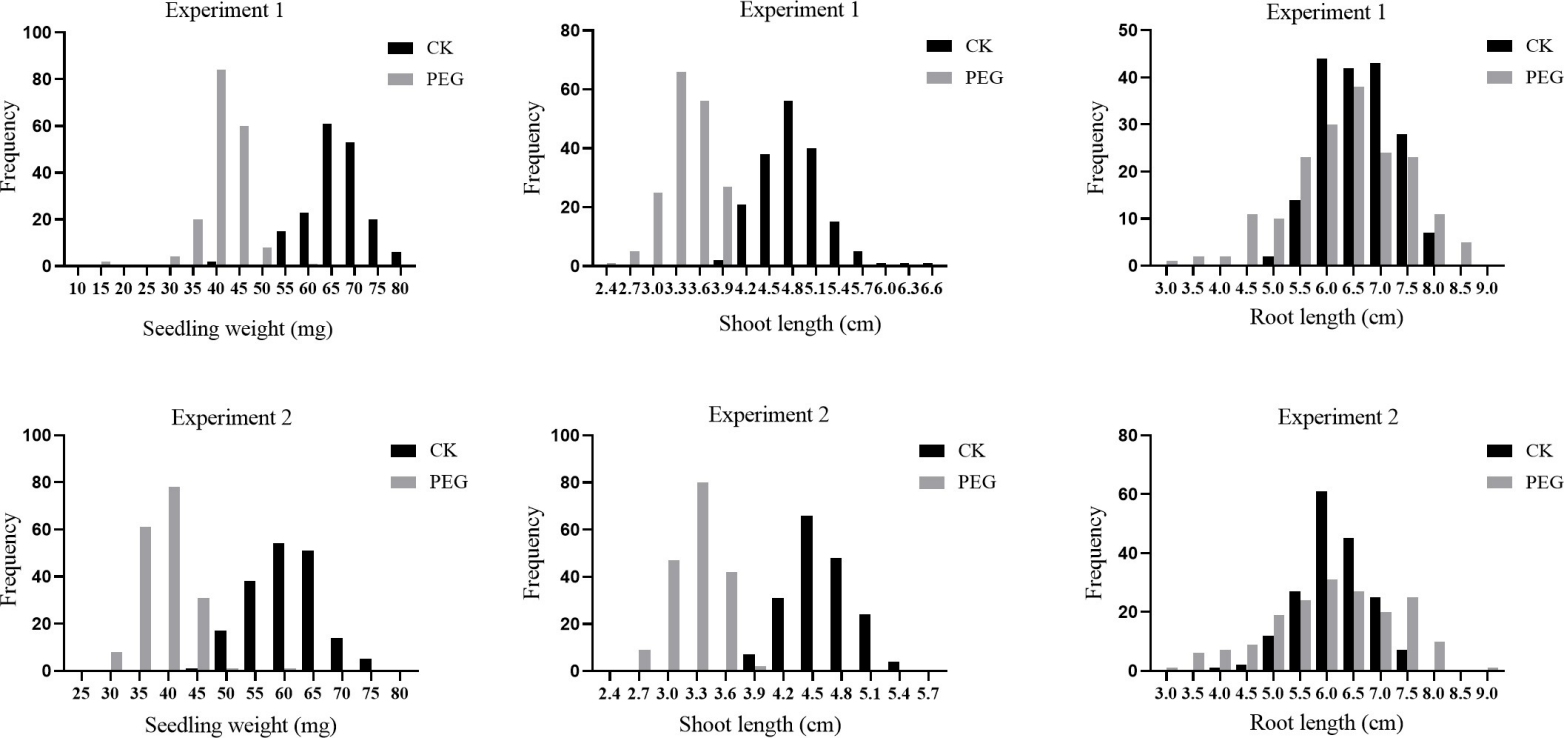
Frequency distributions of Seedling Weight (SW), Shoot Length (SL) and Root Length (RL) under control and PEG stress condition in the RIL population.

The phenotype data analysis showed that each trait varied among two parents and different RILs in both treatments (Fig 3; Table 2). The values of traits root length (RL), shoot length (SL) and fresh seedling weight (SW) of parent JHM were all lower than ZSB to some extent. The phenotypic distributions of mean showed continuous variations and transgressive segregations on both directions of the parents, suggesting polygenic inheritance of all traits in sesame. In the PEG osmotic condition, mean SL and SW of the RIL population were significant inhibited and reduced by 29.3% and 37.1% respectively when compared with the control condition, whereas the mean RL trait was slightly affected and reduced by 2.7%.

**Table 2:** Phenotypic data and heritability of six traits in parents and RIL population.

Traits	Experiment		ZSB	JHM	RIL mean	RIL range	h^2^
Seedling weight (mg)	1	Control	73.35	62.10	60.41	46.56–76.50	0.63
	2	Control	73.13	62.75	66.36	39.40–79.88	
	1	PEG	40.65	35.20	38.87	29.67–58.08	0.55
	2	PEG	41.95	35.25	40.89	12.50–60.93	
Shoot length (cm)	1	Control	5.04	4.88	4.61	3.77–5.47	0.73
	2	Control	5.22	4.94	4.84	3.93–6.56	
	1	PEG	3.18	3.16	3.27	2.67–3.86	0.71
	2	PEG	3.19	3.09	3.41	2.55–3.98	
Root length (cm)	1	Control	7.48	5.78	6.63	4.94–8.18	0.71
	2	Control	7.46	5.86	6.15	3.91–7.43	
	1	PEG	6.48	3.30	6.34	3.24–8.67	0.88
	2	PEG	6.59	3.49	6.09	3.19–8.75	
Relative seedling weight	1		0.55	0.57	0.64	0.53–0.95	0.34
	2		0.57	0.56	0.65	0.16–1.02	
Relative shoot length	1		0.63	0.65	0.71	0.56–0.87	0.70
	2		0.61	0.62	0.71	0.53–0.91	
Relative root length	1		0.87	0.57	0.96	0.61–1.33	0.75
	2		0.88	0.59	0.97	0.58–1.41	

**Fig 4:**
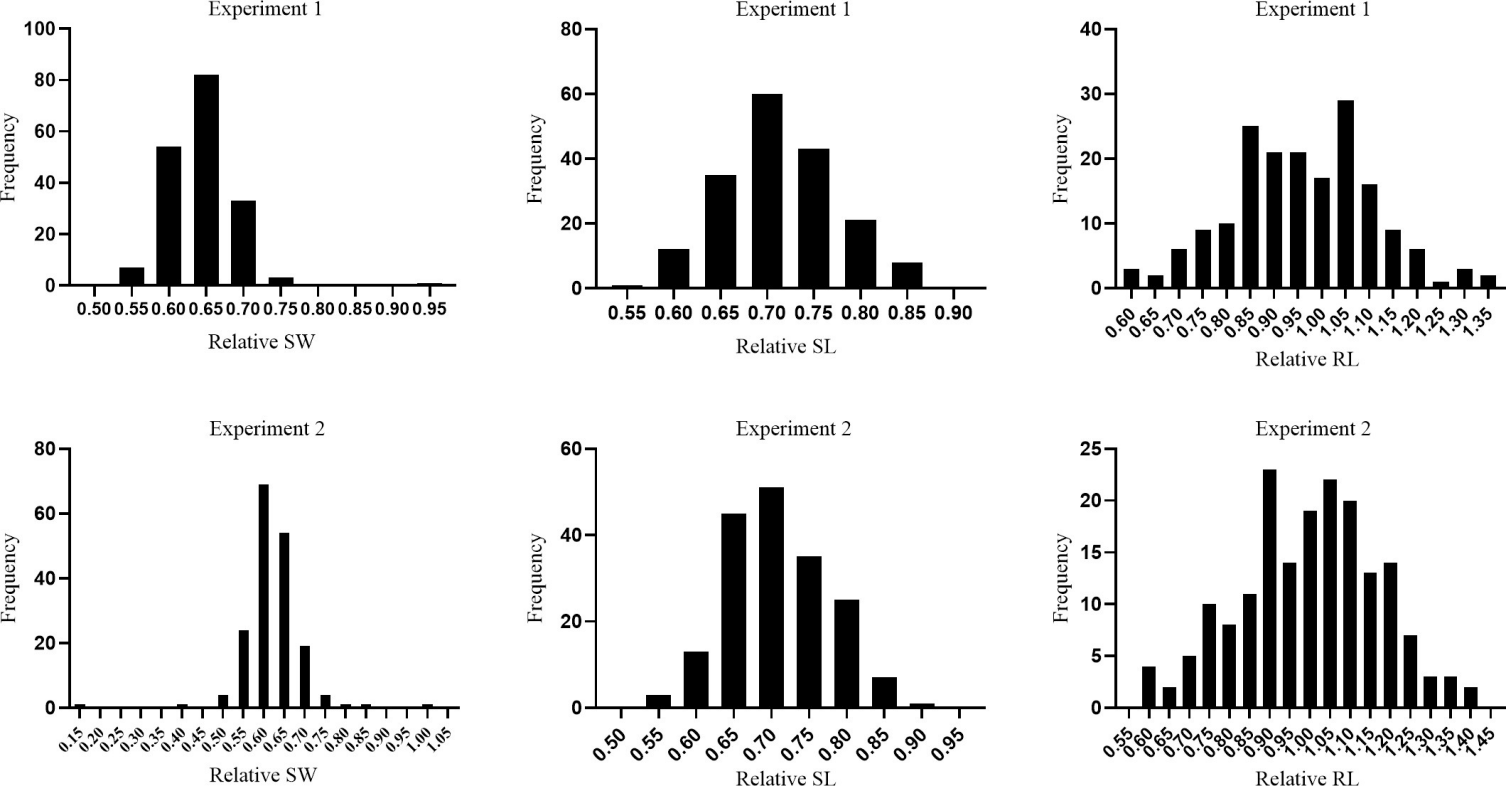
Frequency distributions of relative SW, relative SL and relative RL.

To better understand the responses of these traits to drought stress, we also investigated the relative phenotypic data of these three traits (Fig 4; Table 2). The mean values of relative SW (RSW) and SL (RSL) showed no differences between two parents and mean relative RL (RRL) of ZSB (0.88) was significantly higher than that of JHM (0.58), indicating that JHM was more sensitive to drought than ZSB at early seedling stage. In RILs, highly significant differences were also noted for all three relative parameters.

Among all these nine traits, RLP and RRL had relatively high heritability (88% and 75%), and RRL displayed the most remarkable variation, ranging part from 1 in two directions, indicating the inhibition or induction of osmotic stress on root growth in the population. This also suggested that the responses to drought stress in root of lines were mostly determined by their genotypes. The broad sense heritability of all traits under both conditions ranged from 34% to 88%, with the lowest heritability values recorded for relative seedling weight (34%) (Table 2). Analysis of variance components for the phenotypic data of each trait revealed that effects of genotype for all traits were significant (*P* < 0.01) (S3 Table). The effects of genotype–environment interaction (GEI) were found to be significant for traits SWC, SLC, RLC, SWP, RSW, RSL and RRL, but not significant for traits SLP and RLP. The proportions of GEI mean square to total mean square for RSW, SWC and SWP were relatively larger than that for other traits (S3 Table), suggesting larger influence of GEI on the expression of traits SW under both control and osmotic stress conditions than that of SL and RL in our study.

Correlations among seedling weight, shoot length and root length in both conditions were also surveyed (Table 3). For control condition, all the three traits (herein abbr. SWC, SLC and RLC, respectively) were positively correlated with each other in the two trials. Correlations among SW, SL and RL under PEG stress condition (herein abbr. SWP, SLP and RLP, respectively) were weaker than that under control condition. RLP were positively correlated with SLP in one or two experiments, but SWP had no significant relationships with RLP (significant at *P* = 0.05) in either of two trials. All the identical traits showed significant correlation between control and stress condition, with the average correlation coefficients ranging from 0.347 for SL to 0.472 for RL.

**Table 3:** Correlation coefficents for six traits in the RIL population.

	SWC	SLC	RLC	SWP	SLP	RLP
SWC	1					
SLC	0.193**	1				
	0.390**					
RLC	0.295**	0.249**	1			
	0.381**	0.475**				
SWP	0.776**	-0.008	0.240**	1		
	0.461**	0.042	0.086			
SLP	0.031	0.303**	0.102	0.091	1	
	0.288**	0.390**	0.218**	0.356**		
RLP	0.001	0.172*	0.454**	-0.099	0.414**	1
	0.117	0.380**	0.489**	0.072	0.509**	

The correlation coefficients from experiment 1 and experiment 2 are shown in the first and second rows, respectively

* and ** indicates significance at *P* = 0.05 and 0.01, respectively.

### QTL mapping

Composite interval mapping was used to identified chromosome regions associated with seedling weight, shoot length and root length under control and PEG stress conditions. In total, 34 QTLs were detected for all the traits under both conditions including the relative traits values, and 13 of them could be detected under two experiments. These stable QTLs were mapped to six chromosomes.

#### QTL identification under control condition

Under control condition, eleven QTLs were identified as being associated with these traits (Table 4). *qSLC1*, which could be identified in both experiments, influenced shoot length, located at chromosome 1, had the highest LOD value, explaining an average of 16.26% of the phenotypic variation. Three chromosome regions showed associated with SW. Among them, *qSWC12* was mapped to chromosome 12 and expressed stably across the trials, contributing to higher SW through JHM alleles. For shoot length, besides *qSLC1*, another two stable QTLs were detected on chromosome 5 and 12. One of them, *qSLC12*, was mapped to the interval (c12b062-c12b072) on chromosome overlapped with the interval of *qSWC12* (c12b069-c12b071) on chromosome 12. *qSLC12* had the second highest LOD value and explained up to 9.38% of phenotypic variation in SL. *qSLC1* gained favourable allele from ZSB, while *qSLC5* and *qSLC12* were associated with increased SL through JHM alleles.

**Table 4:** QTLs identified for drought tolerance traits.

Traits	Chromosome location	Locus	Flanking Markers	Experiment 1 (2018)			Experiment 2 (2019)		
				LOD^a^	AE^b^	R^2^(%)	LOD^a^	AE^b^	R^2^(%)
SW-control	Chr2	*qSWC2*	c02b067-c02b073				3.22	1.71	6.54
	Chr8	*qSWC8*	c08b086-c08b090	3.75	1.62	7.17			
	Chr12	*qSWC12*	c12b069-c12b071	5.56	1.99	10.82	2.98	1.56	6.05
SL-control	Chr1	*qSLC1*	c01b092-c01b100	9.06	-0.14	16.38	8.72	-0.17	16.14
	Chr5	*qSLC5*	c05b087-c05b094	3.05	0.08	5.02	2.87	0.10	4.87
	Chr8	*qSLC8*	c08b065-c08b070	3.54	0.08	5.86			
	Chr12	*qSLC12*	c12b062-c12b072	5.34	0.10	9.06	5.30	0.13	9.38
RL-control	Chr1	*qRLC1*	c01b086-c01b087				5.23	-0.20	10.46
	Chr4	*qRLC4*	c04b040-c04b046	4.91	-0.21	9.12	4.54	-0.19	8.33
	Chr6	*qRLC6*	c06b129-c06b135				4.57	0.18	8.46
	Chr10	*qRLC10*	c10b076-c10b080	4.62	-0.20	8.40			
SW-PEG	Chr1	*qSWP1*	c01b003-c01b010	4.20	-1.23	8.20			
	Chr3	*qSWP3*	c03b116-c03b119				3.16	1.39	6.61
	Chr9	*qSWP9*	c09b031-c09b040				3.57	-1.62	7.50
SL-PEG	Chr1	*qSLP1*	c01b032-c01b035	4.27	-0.07	8.26			
	Chr8	*qSLP8*	c08b055-c08b063	3.06	0.06	5.88	3.04	0.07	5.83
	Chr9	*qSLP9-1*	c09b015-c09b021				3.76	0.10	7.26
	Chr9	*qSLP9-2*	c09b031-c09b033				5.56	-0.12	11.00
RL-PEG	Chr1	*qRLP1*	c01b062-c01b070	4.97	-0.31	7.97	4.36	-0.31	6.77
	Chr6	*qRLP6*	c06b054-c06b060	3.28	0.26	5.15			
	Chr7	*qRLP7*	c07b030-c07b036	4.37	-0.29	6.61	4.14	-0.30	6.40
	Chr12	*qRLP12*	c12b032-c12b036	7.17	-0.38	11.85	8.78	-0.45	14.46
RSW	Chr5	*qRSW5-1*	c05b019-c05b029	3.74	0.01	6.80			
	Chr5	*qRSW5-2*	c05b071-c05b074				3.01	0.02	6.33
	Chr6	*qRSW6*	c06b041-c06b045	4.51	0.01	8.29			
	Chr12	*qRSW12*	c12b061-c12b072				3.56	-0.02	7.47
RSL	Chr1	*qRSL1-1*	c01b035-c01b049				3.56	-0.17	6.39
	Chr1	*qRSL1-2*	c01b109-c01b113	5.37	0.02	9.48	6.67	0.02	12.15
	Chr11	*qRSL11*	c11b044-c11b051	3.74	0.02	6.36			
RRL	Chr1	*qRRL1*	c01b052-c01b063	4.12	-0.04	6.56	3.92	-0.04	6.42
	Chr3	*qRRL3-1*	c03b043-c03b055				3.65	-0.04	5.97
	Chr3	*qRRL3-2*	c03b102-c03b113	4.29	-0.04	6.82			
	Chr7	*qRRL7*	c07b020-c07b028	4.45	-0.04	7.09	3.19	-0.04	5.14
	Chr12	*qRRL12*	c12b032-c12b036	6.26	-0.05	10.26	9.36	-0.07	16.47

^a^LOD: likelihood of the odds.

^b^Additive effect: positive and negtive indicated ZSB and JHM allele produced larger value respectively.

^c^E followed by 1 or 2 designate two independent experiments.

A total of four root length QTLs were detected under control condition. One stable QTL (*qRLC4*) located on chromosome 4 were detected in two trials (Table 4), had an average LOD value of 4.73 and explained at least 8.33% of phenotypic variation. The ZSB allele of *qRLC4* was associated with longer root length.

#### QTL identification under PEG stress condition

Eleven chromosome regions were found to be associated with SW, SL and RL under PEG stress condition (SWP, SLP and RLP) (Table 4). For SWP, two QTLs were identified and mapped on chromosome 3 and 9 (*qSWP3* and *qSWP9*), but none of them could be detected across two trials. Four chromosome regions were detected to be associated with SL. Two of them located on chromosome 9. One QTL located on chromosome 8 (*qSLP8*) were steadily expressed in both trials and explained an average of 5.86% of phenotypic variation. *qSLP8* increased SL under osmotic condition through the ZSB allele.

Of the four QTLs associated with RL under PEG stress condition, three could be identified in two experiments (Table 4). Among them, *qRLP12* had the highest LOD value and strongest effect on RL, explaining up to 14.46% of phenotypic variation. The other two QTLs were located on chromosome 1 (*qRLP1*) and 7 (*qRLP7*), explaining at least 4.36% and 4.14% of phenotypic variation respectively. The ZSB alleles of these three stable QTLs were associated with longer root under PEG stress condition.

#### QTL identification for drought tolerance index

DTI was defined as relative traits value in this study. Twelve chromosome regions were identified to be associated with relative seedling weight (RSW), relative shoot length (RSL) and relative root length (RRL) (Table 4). For RSL, *qRSL1-2* detected in both trials had the strongest effect on RSL, explaining an average of 10.82% phenotypic variation, with LOD scores > 5. JHM contributed the positive alleles for *qRSW5* and *qRSL1-2*. Four QTLs were identified for RSW in one of the trials. No stable QTL was detected for RSW.

**Fig 5:**
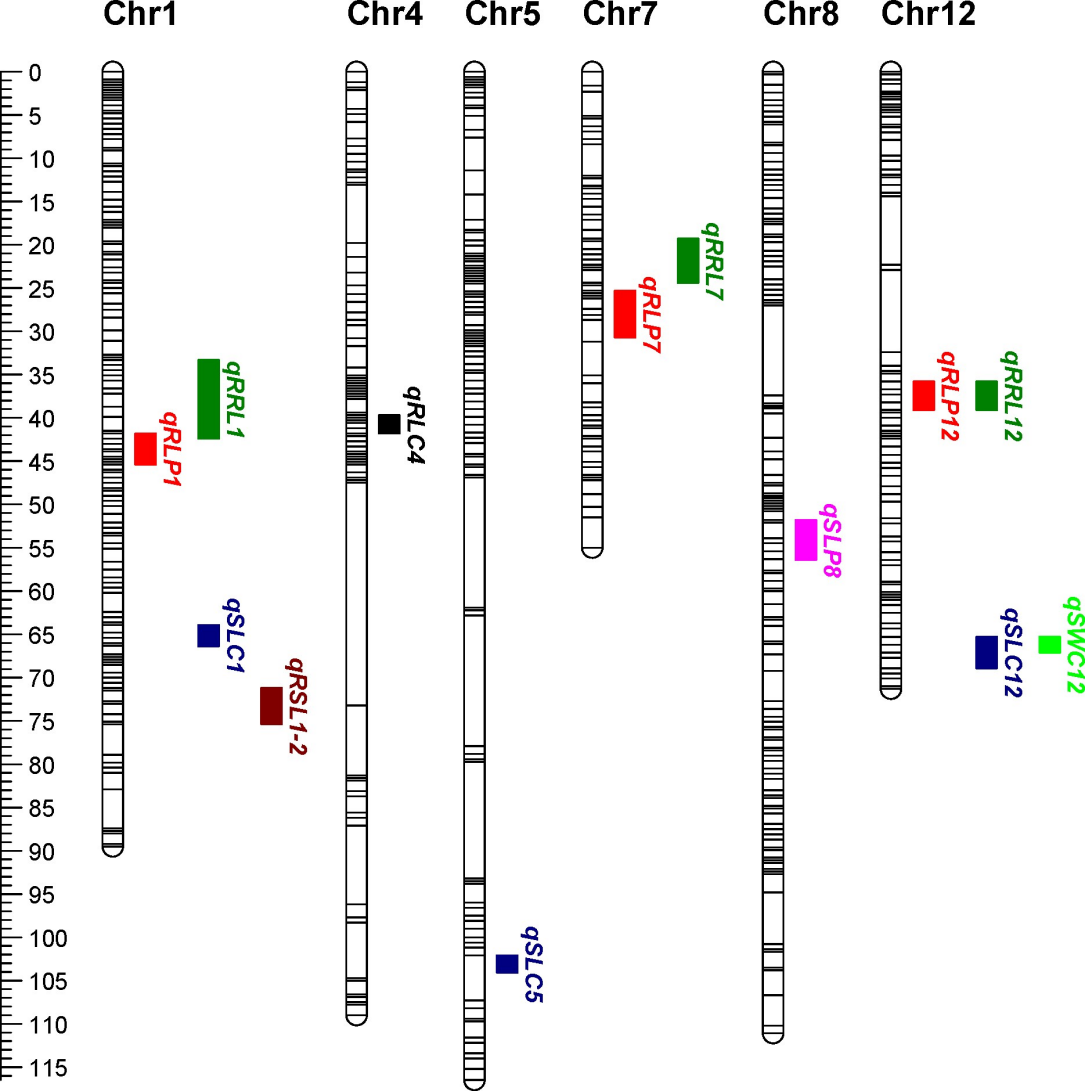
Chromosome location of 13 stable QTLs in the RIL population. Genetic distances are shown at the left in cM. Bars in each chromosome represent bin-markers.

Among the five chromosome regions associated with RRL, *qRRL12* had the strongest effect and explained, on average, 13.37% of the phenotypic variation in two trials, with an average LOD score of 7.81. This QTL was mapped to the same chromosome region as *qRLP12* (Fig 5; Table 4). Besides *qRRL12*, two QTLs, *qRRL1* and *qRRL7*, contributed at least 6.42% and 5.14% of the phenotypic variation in two trials, respectively. All of these three stable QTLs were responsible for the increase of RRL through the ZSB allele.

### Putative candidate genes of major QTLs for drought tolerance

Since the relative traits values, such as RSW, RSL and RRL, reflect the response to drought stress, we identified putative candidate genes of major stable QTLs for drought tolerance. Four genomic regions associated with five major QTLs (*qRSL1-2*, *qRRL1*, *qRRL7* and *qRRL12/qRLP12*) were obtained according to the corresponding flanking markers of each QTL. A total of 465 genes were detected in these four regions, among them, 347 genes were functional annotated while 118 genes were identified as unknown protein, hypothetical proteins or repeat sequences. Variations in the gene region between two parents from the re-sequencing data were also examined.

For RSL, the interval of major QTL *qRSL1-2* contained 92 genes, of which, 74 were annotated as known protein-encoding genes. Four genes, *SIN_1008253*, *SIN_1008230*, *SIN_1008220* and *SIN_1008205*, encoding GTP binding domain, FAD/NAD(P)-binding domain, RNA recognition motif domain and Zinc finger (A20-type) respectively, were homologous to Arabidopsis genes whose functional roles in response to drought stress had been validated through mutagenesis or transgenic studies [30–33]. Two of them, *SIN_1008253* and *SIN_1008220* contained nonsynonymous substitutions in the exons between two parents (S4 Table). *SIN_1008253*, homologous with *AT3G57180*, which encodes a chloroplast localized protein YL1/BPG2 that involved in seedling shoot response to salt stress [31]. Loss of YL1 function caused Na+ accumulation and hypersensitive phenotype of shoot under salt stress [31]. *SIN_1008230* harbored one SNP and a 17bp InDel in the promoter region (at the position of <-1kb from start codon). Overexpressing seedlings of *AT5G67030* (the homologs of *SIN_1008230*) displayed enhanced drought tolerance than the wild-type plants under osmotic stress [32].

For RRL, the physical regions of three major QTLs (*qRRL1*, *qRRL7* and *qRRL12/qRLP12*) on chromosome 1,7 and 12 encompassed 157,161 and 55 predicted genes respectively. Putative genes encoding cellulose synthase [34], eukaryotic translation initiation factor (eIF)-like protein [35], subtilisin-like protease [36], leucine aminopeptidase/peptidase B [37], ELO family member [38], multi antimicrobial extrusion protein [39], aquaporin-like protein [40], WRKY transcription factor [41], AP2/ERF protein [42] and ATP-dependent RNA helicase DEAD-box [43], had been reported to regulate responses to drought stress (S4 Table). Four of these genes comprised nonsynonymous SNPs in the coding region between two parents, including *SIN_1017978* encoding AP2/ERF domain in *qRRL1* region, *SIN_1006102* and *SIN_1011692* encoding eIF-like protein and multi antimicrobial extrusion protein in *qRRL7* region and *SIN_1022428* encoding callose synthase 7 in *qRRL12/qRLP12* region (S4 Table). Two genes, *SIN_1022428* and *SIN_1017975* (within *qRRL1* region), contained variations in their promoter regions.

## Discussion

Sesame is one of the most important oil crops worldwide and provides sorts of specific lignins which are very good for human health. Drought is a major stress effecting sesame growth at early seedling stage. Efforts have been made for drought tolerance QTL and candidate genes identification in recent years [17,18]. However, the drought tolerance related traits are quite complex and controlled by multiple genes and environmental factors. So far, only one genetic study was performed for drought tolerance at sesame seedling stage [17], and no QTL has been reported for root related traits. In this study, 180 RILs were used to identify genomic regions associated with PEG-induced drought tolerance traits, including seedling weight, shoot length and root length at early seedling stage in sesame. Through whole genome re-sequencing, a high-density genetic map was constructed. 13 stable QTLs associated with these traits were detected, and 8 of them were associated with traits related to osmotic stress condition.

In recent decades, with the development of next-generation sequencing (NGS) technology and the release of whole genome sequence of sesame, numerous SNP markers have been used for QTL genetic mapping and GWAS analysis. Bin-maps constructed by high-density SNP markers using NGS have been widely used in sesame QTL mapping [22,44]. In the present study, we re-sequenced each RIL with an average read depth of 4.18× and construct a high-density genetic map with 1354 bins on all 13 chromosomes. The average genetic length was 0.978 cM per bin, which was similar with previous maps [22,44]. Comparing with other sesame genetic maps developed by restriction-site associated DNA sequencing (RAD-seq) or GBS approach, WGRS strategy could acquire much more variants. We obtained 466,911 high-quality SNPs and 72,981 InDels for bin markers assignment, which was more than 30 times of 13,679 SNPs identified by GBS in Zhang et al. [22] and 11,924 SNPs detected by RAD-seq in Zhang et al. [44]. The high-density variants could improve the resolution of QTL and also help perform fine mapping of QTL controlling various agronomic traits.

For drought tolerance evaluation, due to the unpredictability of rainfall and soil heterogeneity of field experiment, PEG solution has been widely employed as an alternative to simulate water shortage condition, especially for the trials at germination and seedling stage [4,7,13,17]. In sesame, Li et al. used relative values of germination rate and fresh weight (the ratio of the traits value under stress conditions to the same traits under stress free conditions) to evaluate the drought tolerance of sesame at early seedling stage [17]. For drought tolerance at sesame flowering stage, Dossa et al. investigated wilting level of the whole plant, stem length, and some yield related traits [18]. However, no comprehensive genetic study has been conducted yet for the root traits under desiccation stress condition in sesame. In this study, three characters including SW, SL and RL were measured in the RIL population under control and PEG stress condition. All the measured traits showed significant genetic variation under both conditions, which enable the identification of QTLs associated with traits in sesame. We found high level of broad heritability of the trait RL under stress condition, indicating the selection of this trait could be effective in the breeding program. Relative performances of trait values were usually used as indicators for the evaluation of stress tolerance. We also investigated relative SW, SL and RL in current study and the results showed that relative RL had the highest heritability among these three indexes, and exhibited the most remarkable variation in RILs, distributing part from 1 in both directions, suggesting the PEG solution treatment in our study only inhibited the root growth of some lines. This also indicated that the different performances of root length in the RILs mainly depended on genotypic variation. Analysis of variance components for these traits revealed that GEI effect was significant for all traits except SLP and RLP. Relatively high proportions of GEI mean square were found for SWC, SWP and RSW, which lowered their heritability estimates. These indicated that SW may be more affected by environment than other two traits.

In this study, QTLs were identified for all the traits under control and osmotic stress conditions. For root length, we found that in ZSB, both *qRLP12* and *qRRL12* were mapped to the same interval on chromosome 12, associated with increased RL under PEG stress condition and RRL. *qRLP12* and *qRRL12* had the highest LOD values of all the QTLs associated with RLP and RRL respectively, and explained up to 14.46 and 16.67% of the phenotypic variation. These results implied that this interval on chromosome 12 contains a major QTL that associated with root length and drought tolerance. To our knowledge, this is the first report of QTLs that associated with root length in sesame. Since the root is the tissue responsible for water uptake, deep root system can help plants absorbing water from deep soil layers to avoid drought stress. This interval in ZSB could be useful for drought tolerance breeding in sesame. In addition to *qRLP12*, the interval of other two stable QTLs controlling root length under osmotic stress condition were both closely linked with that of QTLs associated with relative root length (*qRLP1* and *qRRL1*, *qRLP7* and *qRRL7*) (Fig 4). This strongly indicated close relationship between traits RLP and RRL, which may enable selection for the complex drought tolerance trait through an easily observable related trait, such as relative root length and root length under stress condition.

The traits of above ground part also play important role for avoiding drought. Long shoot is conducive to rapid seedling formation and allows deeper sowing. In this study, eight QTLs were identified for SL under both conditions, four of them (*qSLC1*, *qSLC5*, *qSLC12* and *qSLP8*) stably expressed in two trials. For SW, only one major QTL *qSWC12* for SW under control condition was detected in two trials. We could not detect any stable QTL for trait SW under osmotic stress condition and trait RSW, which may be due to their relative low heritabilities. This indicated that seedling weight may not be suitable as selection trait for drought tolerance in seedling stage of sesame. *qSWC12* and *qSLC12* was both located on similar region of chromosome 12. QTLs for shoot length and seedling weight co-location on chromosome have also been reported in soybean [45]. The JHM alleles for *qSWC12* and *qSLC12* were related to increased SW and SL under control condition, indicating the parent with lower phenotypic values may also contain favorable alleles for traits SW and SL. QTL cluster in this region could be raised by both linkage and pleiotropic effects.

The positions of mapped QTLs in this study were also compared with previous drought tolerance related loci. Li et al. detected nine and 15 QTLs for drought and salt tolerance indexes by utilizing germination rate and fresh seedling weight as phenotypes through GWAS at germination stage [17]. None of the loci associated with fresh seedling weight were located in the QTL regions identified in our study, but a QTL *qSGR6*.*1* related to germination rate index under salt stress shared the same location as *qRRL12*. Thabet et al. detected several SNPs associated with RL_DTI and germination percentage_DTI on barley chromosome 3H and 4H were very close to each other at the corresponding regions [46]. Whether the interval of *qRRL12* were associated with germination rate in our population need further experiments. Dossa et al. identified four genomic regions associated with five traits including stem length and yield related traits at flowering stage of sesame with GWAS [18]. All of them were located at the positions different from those of QTLs in our study, suggesting various genetic mechanisms of drought tolerance may exist between the germination and flowering stages.

Candidate genes located within the genomic region of four major QTLs for drought tolerance were also identified in this study. For trait RSL, we found three genes in *qRSL1-2* region may be putative candidates according to the previous studies of their homologs in *A*. *thaliana* [31–33]. It is noteworthy that one of them, *SIN_1008253*, which contained seven nonsynonymous SNPs in the exons between two parents, is homologous with a well-studied *A*. *thaliana* gene *YL1* (*AT3G57180*) that influenced seedling shoot growth under salt stress [31]. *YL1* encodes a YqeH-type GTPase that is conserved in plant chloroplast and regulate the shoot response to salt stress through *ABI4* [31]. *SIN_1008230*, contained variations in its promoter region between parents, is homologous with a zeaxanthin epoxidase gene in *A*. *thaliana* (*AtZEP*, *AT5G67030*) that is an important enzyme in ABA biosynthesis. ZEP-overexpressing plants showed more developed rosette leaves and lateral roots than wild-type plants under mannitol stress condition [32]. Another gene *SIN_1008220*, encoding a peptide containing RNA recognition motif domain. Its homologs in *A*. *thaliana* (*PSRP2*, *AT3G52150*) is a chloroplast-localized ribosomal protein that played a role as a negative regulator on seedling growth under salt stress condition [33].

For trait RRL, five putative candidate genes located on three major QTLs regions (*qRRL1*, *qRRL7* and *qRRL12/qRLP12*) were identified in this study. *SIN_1022428* was the only one gene which harbored variations that caused peptide change among all 55 annotated genes in *qRRL12/qRLP12* region. The *A*. *thaliana* homolog (*AT1G06490*) of *SIN_1022428* encodes callose synthase 7, which evolves in callose deposition in developing sieve elements during phloem formation. The 5-day-old seedling of *AT1G06490* mutant developed much shorter roots than wild-type [34]. Two genes, *SIN_1017975* and *SIN_1017978*, located at the interval of *qRRL1*, were annotated as WRKY transcription factor and AP2/ERF protein, whose homologs in *A*. *thaliana* were closely related to abiotic stress responses [41,42]. *SIN_1017978*, also named as *AP2si16*, was reported to be highly induced by drought stress condition in previous study [47]. In the region of major QTL *qRRL7*, *SIN_1006102* and *SIN_1011692* which encode eIF-like protein and multi antimicrobial extrusion protein were the two genes included synonymous SNP. *SIN_1006102* was homologous with *AT5G57870* that was proved to be involved in the responses to dehydration, salinity, and heat stress through mutagenesis analysis [35], while *SIN_1011692*-encoding protein shared high similarity with a detoxification efflux carriers (DTX)/multidrug and toxic compound extrusion (MATE) transporter *AT5G65380* (*DTX27*). Overexpression of a cotton homolog of *DTX27* could enhance drought, salt, and cold stress tolerance in transgenic Arabidopsis plants [39]. The confirmation of candidate genes needs further fine mapping of these QTLs and functional validation experiments.

## Conclusions

In conclusion, we performed first QTL mapping of drought tolerance related traits using a RIL population and identified root length QTLs for the first time in sesame. By using PEG treatment, inheritances of three traits including seedling weight, shoot length and root length were interpreted. Root length had the largest broad sense heritability, while seedling weight occupied the lowest position. Using WGRS technique, we identified a total of 13 stable QTLs for seedling weight, shoot length and root length at seedling stage of sesame. Eight of them were associated with traits under water stress condition. Four of them explained more than 10% phenotypic variation and had an LOD score larger than 6. The current study was the first report for QTL identification for root length, which is considered as one of the most important traits for drought tolerance in plant. The major QTL regions as well as the associated candidate genes and linked markers can provide potential genetic resources for molecular marker-assisted selection and further cloning of functional genes for drought tolerance in sesame.

## Supporting information

S1 Fig Collinearity analysis of genetic map with the sesame reference genome Zhongzhi No. 13. (TIF)

S1 Table Distribution of total filtered SNPs and InDels on 12 chromosomes. (XLSX)

S2 Table The information of high-density bin map. (XLSX)

S3 Table Mean Squares (MS) and estimated variances (VA) of all traits under control and osmotic stress conditions across two environments. (XLSX)

S4 Table Putative candidate genes in the interval of major QTLs for drought tolerance. (XLSX)
