# Supplementary figures and images for: QTL mapping of PEG-induced drought tolerance at the early seedling stage in sesame using whole genome re-sequencing
Source: PLoS One. 2021 Feb 24;16(2):e0247681. doi: 10.1371/journal.pone.0247681 (PMC7904189; doi:10.1371/journal.pone.0247681)

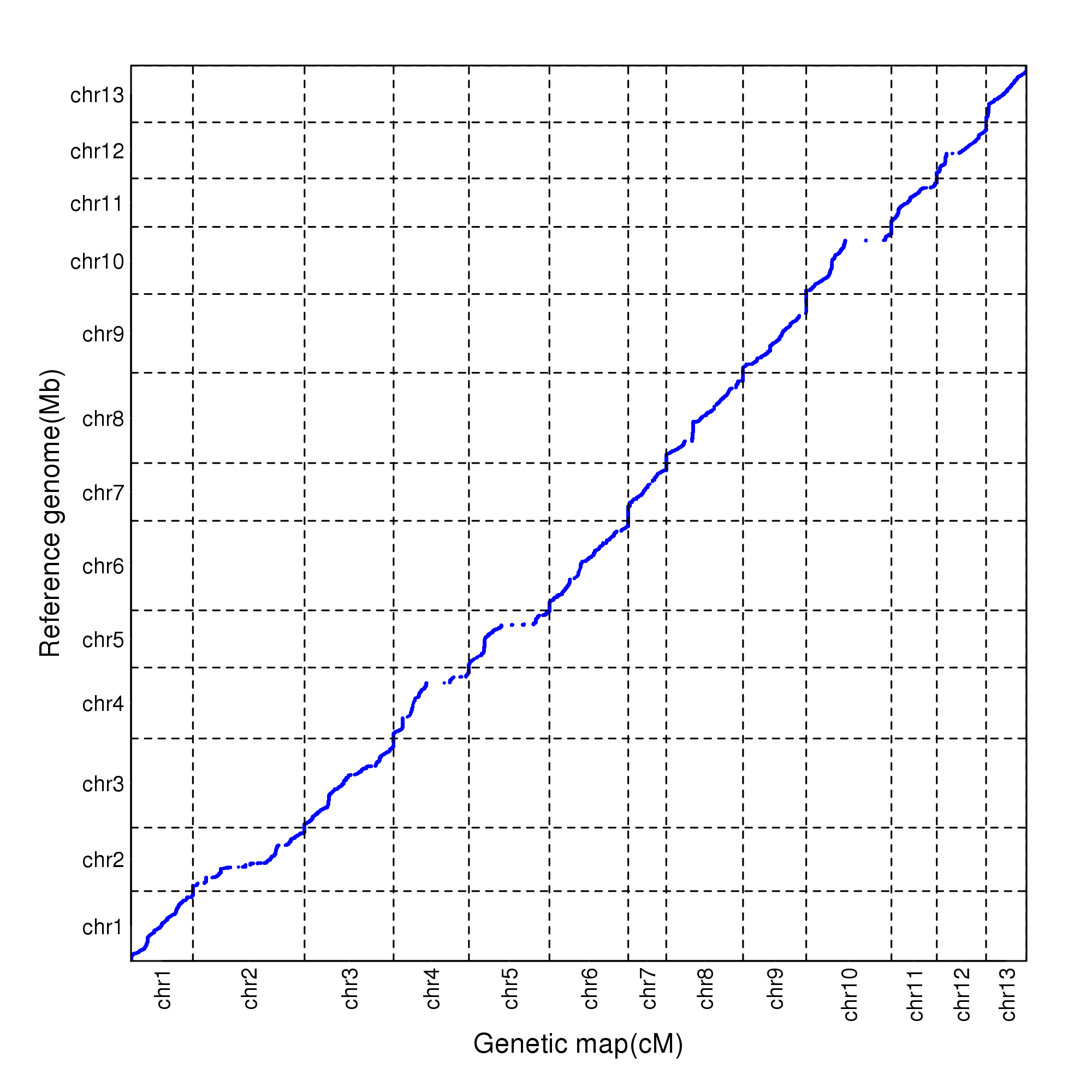

Supplement: S1 Fig — (TIF) [file pone.0247681.s001.tif]
